# Corrigendum: Modeling dynamic interactions between pre-exposure prophylaxis interventions & treatment programs: predicting HIV transmission & resistance

**DOI:** 10.1038/srep13037

**Published:** 2015-09-18

**Authors:** Virginie Supervie, Meagan Barrett, James S. Kahn, Godfrey Musuka, Themba Lebogang Moeti, Lesego Busang, Sally Blower

Scientific Reports 1: Article number: 18510.1038/srep00185; published online: 12072011; updated: 09182015

The original version of this Article contained a typographical error in the spelling of the author Lesego Busang which was incorrectly given as Lesogo Busang. This has now been corrected in the PDF and HTML versions of the Article.

